# *Leishmania* RNA virus: when the host pays the toll

**DOI:** 10.3389/fcimb.2012.00099

**Published:** 2012-07-12

**Authors:** Mary-Anne Hartley, Catherine Ronet, Haroun Zangger, Stephen M. Beverley, Nicolas Fasel

**Affiliations:** ^1^Department of Biochemistry, University of LausanneEpalinges, Switzerland; ^2^Department of Molecular Microbiology, Washington University School of MedicineSt. Louis, USA

**Keywords:** *Leishmania*, *Totiviridae*, mucocutaneous leishmaniasis, dsRNA virus, toll-like receptor

## Abstract

The presence of an RNA virus in a South American subgenus of the *Leishmania* parasite, *L. (Viannia)*, was detected several decades ago but its role in leishmanial virulence and metastasis was only recently described. In *Leishmania guyanensis*, the nucleic acid of *Leishmania* RNA virus (LRV1) acts as a potent innate immunogen, eliciting a hyper-inflammatory immune response through toll-like receptor 3 (TLR3). The resultant inflammatory cascade has been shown to increase disease severity, parasite persistence, and perhaps even resistance to anti-leishmanial drugs. Curiously, LRVs were found mostly in clinical isolates prone to infectious metastasis in both their human source and experimental animal model, suggesting an association between the viral hyperpathogen and metastatic complications such as mucocutaneous leishmaniasis (MCL). MCL presents as chronic secondary lesions in the mucosa of the mouth and nose, debilitatingly inflamed and notoriously refractory to treatment. Immunologically, this outcome has many of the same hallmarks associated with the reaction to LRV: production of type 1 interferons, bias toward a chronic Th1 inflammatory state and an impaired ability of host cells to eliminate parasites through oxidative stress. More intriguing, is that the risk of developing MCL is found almost exclusively in infections of the *L. (Viannia)* subtype, further indication that leishmanial metastasis is caused, at least in part, by a parasitic component. LRV present in this subgenus may contribute to the destructive inflammation of metastatic disease either by acting in concert with other intrinsic “metastatic factors” or by independently preying on host TLR3 hypersensitivity. Because LRV amplifies parasite virulence, its presence may provide a unique target for diagnostic and clinical intervention of metastatic leishmaniasis. Taking examples from other members of the *Totiviridae* virus family, this paper reviews the benefits and costs of endosymbiosis, specifically for the maintenance of LRV infection in *Leishmania* parasites, which is often at the expense of its human host.

## Introduction

**Figure 1:**
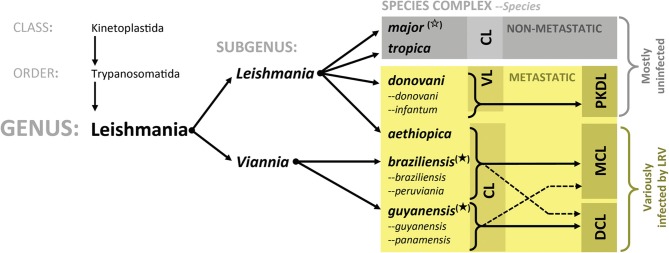
**Leishmanial phylogeny and LRV presence.** Intrinsic parasite factors underlying clinical disparities in metastatic leishmaniasis. Species listed are those relevant to this text. The *L. mexicana* species complex (of the *L. leishmania* subgenus) was omitted for simplicity; thus far, no LRV has been reported in their member species. **CL**, Cutaneous leishmaniasis; **DCL**, Disseminated cutaneous leishmaniasis; **VL**, Visceral leishmaniasis; **PKDL**, Post Kala Azar dermal leishmaniasis; **MCL**, Mucocutaneous leishmaniasis; **LRV**, Leishmania RNA virus; (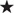), LRV found in numerous isolates within group; (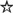), LRV found only in a single, perhaps exceptional, isolate in this group. **Non-starred:** groups in which LRV infection has not been reported.

Endemic in 98 countries, leishmaniases are caused by various species of the *Leishmania* protozoan parasite and exhibit a wide spectrum of clinical manifestations, ranging from a cutaneous lesion (CL) to a fatal visceralization of disease (VL) (Kaye and Scott, 2011; Alvar et al., 2012). Parasites are transmitted through the bite of a sand fly vector, establishing infection in a local CL, although asymptomatic infections are not uncommon. Some cases develop latently, reactivating later as a disseminated or metastatic infestation, complicating clinical outcome, and known to be refractory to standard therapeutic intervention. In South America, up to 10% of CL cases progress to mucocutaneous disease (MCL) forming destructive secondary lesions in the mucosa of the mouth and nose and even occurring in those with asymptomatic primary infections. The risk of this clinical complication can be considered as a distinguishing trait of the *Leishmania (Viannia)* subgenus, as it is mainly caused by species within the group (predominantly *L. braziliensis* but also *L. guyanensis* and *L. panamensis*). Importantly, the clinical presentations of metastatic disease differ between *Leishmania* species (Figure 1). For example, while *L. braziliensis* and *L. panamensis* conform to the CL-to-MCL dissemination pattern described above, *L. guyanensis* gives rise less frequently to mucosal lesions [although reported (Guerra et al., 2011)] and instead more often result in chronic disseminated cutaneous leishmaniasis (DCL) with no reported anatomical specificity. Whatever the individual outcome, the general propensity toward infectious metastasis in South America seems to rely on an intrinsic parasite factor of the *Viannia* subgenus, where species-specific features underlie tissue-specific divergences. A shared feature among the metastatic *Leishmania* is their degree of dormancy and chronicity, as reactivation and dissemination is often only developed months or even years after the initial infection (Marsden, 1986; Ronet et al., 2010). This infective resurgence has been largely attributed to factors extrinsic to the parasite, such as the host environment and its genetic susceptibility, and it is proposed that virulent parasites are selected, kept dormant, and then later revived under immunosuppressed or stressed conditions. For example, antimony treatment during primary infection has been implicated in the development of MCL (Saravia et al., 1990; Arevalo et al., 2007; Souza et al., 2010). It is important to note, however, that dissemination is not completely unique to the *Viannia* subgenus. The *donovani* species complex (of the *Leishmania* subgenus) shows a reversed symptomatic kinetic: visceralizing during a primary infection known as Kala Azar with a risk of later reactivating as a disseminated cutaneous infestation (Post Kala Azar Dermal Leishmaniasis, PKDL) (Figure 1). Still, the metastatic proclivity in these parasites is probably quite different from that seen in MCL and the factors underlying visceralizing tropism in *L. donovani* are purportedly encoded in species-specific genes (Zhang et al., 2008; Zhang and Matlashewski, 2010).

A common thread running through most cases of metastatic infection is the onset of a destructive hyper-inflammatory immune response that is characterized by a deluge of activated immune cells, swelling, and destroying local tissue (Marsden, 1986; Ronet et al., 2010). This overreaction is very likely instigated by a parasite factor. Indeed, the intrinsic *Leishmania* virus of *L. guyanensis* was recently shown to exacerbate inflammation and may prove to be a major driver of metastatic potential in *L. (Viannia)* parasites (Ives et al., 2011; Ronet et al., 2011). Although *Leishmania* viruses have been identified in major metastatic strains of *L. braziliensis* and *L. guyanensis*, metastasis can occur in absence of LRV, such as is the case for *L. panamensis*. Thus, LRV may have a variable contribution to this phenotype, acting alone or in concert with other factors, such as the host genetic background or species-specific parasite virulence factors. In this report, we review the current knowledge on *Leishmania* virus and its interaction with the host immune system in an effort to gauge its clinical impact and potential use in the diagnosis and treatment of disseminated leishmaniases.

## New world leishmaniasis and *leishmania* RNA virus

### Parasite factors underlying disease phenotype

While the role of host factors cannot be overlooked, parasite pedigree is still the most reliable predictive tool of disease phenotype, implying that heritable parasite factors are the major determinants of clinical variation. Yet, despite the considerable clinical differences in leishmaniases across the *Leishmania* phylogenic tree (Figure 1), unique genes between species are relatively scarce (Ivens et al., 2005; Peacock et al., 2007; Smith et al., 2007). Analysis of reference genomes for *L. major, L. mexicana, L. infantum, and L. braziliensis* have further confirmed the low number of species-specific genes, albeit that variation amongst homologs is considerable (Rogers et al., 2011). Nevertheless, no obvious patterns emerge from this variation, which sufficiently explain the symptomatic groupings of certain species. *L. braziliensis* stands out for having a high degree of single nucleotide polymorphisms and “lost” genes, which could, in conjunction with the presence of its 67 unique genes, be the reason for its metastatic tropism and increased virulence (Rogers et al., 2011). Of particular interest are genetic differences involved in the control of oxidative stress. Being an intracellular infection, oxidative destruction in the phagolysosome is a major mechanism of parasite elimination. Avoidance of this killing may be a way of developing latency and later, metastasis. *L. braziliensis* is known to carry supplementary copies of NADPH-dependent fumarate reductase and a homolog of a glutathione peroxidase (as well as having lost a trypanothione synthase-like protein) although it is not yet known whether these enzymes influence the sensitivity of *L. braziliensis* to oxidative stress. The sequencing of *L. guyanensis* and *L. panamensis* genomes will add valuable information to the genetic determinants of oxidative resistance and their contribution to metastatic virulence. Nevertheless, these genetic differences are still not sufficiently predictive or explanatory for the diversity of pathology. Instead, divergence in clinical outcome could be related to differential protein expression achieved through changes in gene regulation, copy number, or the presence of pseudogenes (Lynn and McMaster, 2008; Depledge et al., 2009; Rogers et al., 2011).

In light of LRV infection, parasitic genes controlling RNA-mediated interference (RNAi) are also of interest. The nucleic acid of LRV is potentially recognized by this parasite defense mechanism targeting foreign RNA. While *Leishmania* are not known to express RNA sensors such as those seen in mammals (PKR, RIG-I, MDA-5), some *Leishmania* species express a potent RNAi activity (Lye et al., 2010). Functional RNAi machinery is mostly absent in the *L*. *Leishmania* subgenera (*L. major, L. donovani, L. mexicana*) but has been retained in the major metastatic parasites of the *L. (Viannia)* subgroup (*L. braziliensis, L. panamensis*, and *L. guyanensis*) (Lye et al., 2010). Correspondingly, LRV1 has been found in *L. braziliensis* and *L. guyanensis*, although thus far not in *L. panamensis* (which has been less thoroughly examined). The sole exception to this association is the presence of LRV2 occurring in a single isolate of *L. major* (Scheffter et al., 1995), a Leishmania species a functional lacking RNAi (Lye et al., 2010). Variability in RNAi efficiency between evolutionary lines is also found in many other organisms (playing a strong role in *Drosophila* and *C. elegans*, but minimally functional in mammals. Further studies are needed to define whether retention or losses of RNAi are related to the evolution of viral interaction as is hypothetically exemplified in the co-maintenance of RNAi and LRVs.

### LRV: a member of the *totiviridae* family

**Figure 2:**
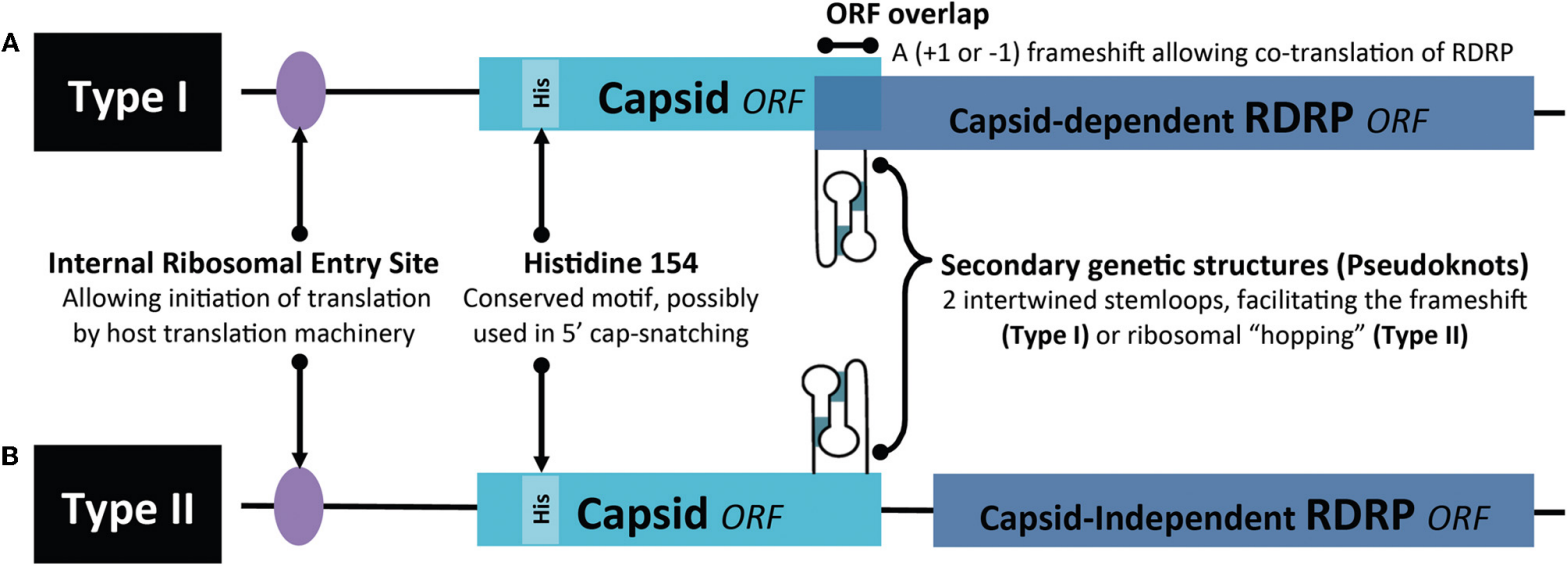
**Organization of the *Totiviridae* genome. (A)** Type I represents the classic capsid-dependent organization with overlapping ORFs, where a −1 or +1 frameshift (under the control of a pseudoknot structure) allows for translation of the RDRP fused to the capsid. **(B)** Type II hosts a capsid-independent RDRP ORF. ORF, open reading frame; RDRP, RNA-dependent-RNA-polymerase; IRE, Internal ribosomal entry site.

*Leishmania* viruses are classified in the *Totiviridae* family (Patterson, 1990; Weeks et al., 1992) encompassing non-enveloped, icosahedral particles present in protozoa [*T. vaginialis* and *G. lamblia* (Wang and Wang, 1991)], yeast (Wickner, 1996), fungi, plants, arthropods (Wu et al., 2010; Zhai et al., 2010; Isawa et al., 2011), penaeid shrimp (Poulos et al., 2006) and even vertebrates [salmon (Lovoll et al., 2010)]. The 40 nm viral particle is composed of a non-segmented dsRNA genome between 4 and 8 kb in length encoding a major capsid protein and a capsid-RNA-dependent RNA polymerase (RDRP) fusion protein, essential for the replication of the dsRNA virus (Figure 2A). This RDRP has, however, been observed as independent from the capsid protein (Figure 2B), for example in the myonecrosis virus infecting penaeid shrimp (Poulos et al., 2006) and the fungal virus, *Helminthosporium victorivirus* (Huang and Ghabrial, 1996). Some totiviruses have additional proteins encoded in their RNA genome, such as the antifungal killer toxin that was used to protect maize against corn smut (Allen et al., 2011). LRV seems to follow the generic totiviridae conformation described above, albeit for gene arrangement and sequence variation between LRV1 and LRV2, described below.

While virus-like particles were described in *Leishmania hertigi* in 1974 (Molyneux, 1974), the first molecular description of *Leishmania* RNA virus came only in the subsequent decade for the two *L. guyanensis* strains: MHOM/SR/81/CUMC1A (Tarr et al., 1988; Stuart et al., 1992) and MHOM/BR/75/M4147 (Widmer et al., 1989) then later in *L. braziliensis* (Salinas et al., 1996). The sole reported LRV found outside *Viannia* was identified in the *L. major* strain (MHOM/SU/73/5-ASKH) (Scheffter et al., 1995). These have been separately categorized as LRV1 and LRV2 in *L. (Viannia)* and *L. major*, respectively, due to their significant sequence differences. Phylogenetic studies on LRVs showed that the genetic distances between LRV1 and LRV2 are similar to those between each parasite strain and that this similarity was further clustered according to geographical origin of the parasite (Scheffter et al., 1995; Widmer and Dooley, 1995). Thus, the viruses were present in *Leishmania* parasites prior to the New/Old World divergence and seem to have co-evolved with their *Leishmania* host (Scheffter et al., 1995; Widmer and Dooley, 1995). Unlike LRV1 of *L. (Viannia)*, the relationship of LRV2 in *L. major* to disease severity or alteration in clinical phenotype has not yet been explored.

Thus far, no dsRNA viruses have been identified in *Leishmania's* protozoan contemporaries such as *Trypanosoma brucei*, *T*. *cruzi* or *Plasmodium*. The most studied members of the *Totiviridae* family are the two dsRNA L-A and L-BC viruses infecting *S. cerevisae* (Wickner, 1996), where studies focus on their relation to the host and modulation of gene expression. As determined in *S. cerevisae*, the plus-ssRNA is synthesized on a dsRNA template by RDRP. Interestingly, these viral ssRNAs lack the 5′ cap structure and are not polyadenylated, features essential for mRNA stability and efficient translation. It has been suggested that this vital 5′ cap could be pirated from host RNA by a unique mechanism dubbed “cap-snatching”. Here, the 5′ m^7^Gp of host mRNA is transferred onto the diphosphorylated 5′ end of the viral transcripts (Fujimura and Esteban, 2011). The viral capsid plays a central role in this theft, where a histidine at position 154 in the protein has been deemed essential. Further, the presence of a trench on the capsid's outer surface [identified by crystallography (Naitow et al., 2002)] is reminiscent of those described for yeast guanyltransferases suggesting convergent evolution (Fujimura and Esteban, 2011). Cap-snatching could also be carried through to fungal totiviruses, as evidenced by some conserved amino acids in the proposed catalytic cleft (Fujimura and Esteban, 2011). It is not known whether this type of capping is also relevant in other totiviruses or if they rather use the “decapping” mechanism previously proposed for yeast L-A viruses (Tang et al., 2005) where unprotected viral mRNAs are shielded from host cell exonucleases by allowing translation of the viral ssRNA through an internal ribosomal entry site (Masison et al., 1995). Besides mRNA cap-theft, the translational toll of the virus on the host cell can be extraordinarily taxing. In yeast, viral capsid has been shown to comprise several percent of total host protein, producing at least 1000 particles per cell, each particle consisting of about 120 capsid proteins (but only 1 or 2 capsid-RDRP fusion polypeptides).

Translation of viral ssRNA has been best described in yeast. Here, *Totiviridae* translation takes place in the host cytoplasm producing the capsid and in most instances, a capsid-RDRP fusion polypeptide. This latter protein is obtained through an inefficient −1 or +1 frameshift, which is under the control of an RNA pseudoknot structure placed upstream of the capsid gene stop codon (Figure 2). N-terminal acetylation of the capsid protein is essential for viral assembly with the capsid being, in turn, important for the packaging of the RDRP and viral genome into the particle, whereas the polymerase domain of RDRP is required for capturing the ssRNA viral molecule (Ribas and Wickner, 1998; Fujimura and Esteban, 2011). Motifs important for polymerase function are conserved among the RDRP sequences of different members of the Totiviridae (Maga et al., 1995b; Routhier and Bruenn, 1998), suggesting similar mechanisms for their transcription.

Similarly to other totiviruses, LRV exists predominantly as a 5.3 kb double-stranded RNA within its capsid, having a plus-strand mRNA for viral polypeptide synthesis (Weeks et al., 1992). The *L. guyanensis* viral particles were demonstrated to have RNA polymerase activity, essential for the replication of RNA viruses (Widmer et al., 1990). Comparison of the two genomes revealed a consensus nucleotide sequence of 5283 base pairs with an overall 76% sequence identity (Scheffter et al., 1994). Each guyanensis virus was given a different designation, namely LRV1-1 (for the virus of the *L. guyanensis* CUMC-1) and LRV1-4 (*L. guyanensis* M4147) although a revision of this nomenclature is currently in preparation. Further analysis of LRV1 sequences identified 3 open reading frames (ORFs) on the plus-strand of LRV1-1 while 4 ORFs were identified in LRV1-4. In both cases, ORF2 and ORF3 are known to encode the major viral proteins of the capsid and capsid-RDRP. Similar to other totiviruses, this RDRP is formed as a fusion protein by a +1 ribosomal frameshift (Maga et al., 1995a; Lee et al., 1996; Ro et al., 1997b; Kim et al., 2005). The predicted protein sequences of the other ORFs have, so far, shown no significant homology with known proteins or any evidence of encoding polypeptides (Stuart et al., 1992; Scheffter et al., 1994). In the case of LRV-2 (the exceptional LRV occurrence in “Old world” leishmanial parasites), the site encoding RDRP (ORF3) is predicted not to be overlapping that of the capsid (ORF2) but is rather separated by a single codon and therefore could be encoded in an independent ORF. Here, translation of the RDRP gene in LRV-2 could be driven by the presence of an additional internal ribosomal entry site upstream of ORF3 or via the action of a pseudoknot structure participating in ribosomal “hopping,” as proposed by Scheffter (Scheffter et al., 1995). These latter mechanisms could be relevant even for those LRV genomes, which do have overlapping ORFs, but the extent to which these are functioning have not yet been resolved. Alternatively, we could postulate that RDRP is synthesized from a trans-spliced mRNA, as done for mRNAs in trypanosomatids. However, such a trans-splicing mechanism would generate mRNAs of smaller sizes (not observed thus far) and would hinder the production of capsid-RDRP fusion proteins.

The fact that, in *Leishmania*, mRNAs are matured by the addition of m7GpppX through trans-splicing of a capped 39-nucleotide mini-exon sequence, suggests that translation of parasitic (non-viral) ssRNA could rely on internal ribosomal entry (Zamora et al., 2000). Interestingly, an internal ribosomal entry site has been mapped in the LRV-1 genome and would potentially allow for the translation of uncapped viral ssRNA (Maga et al., 1995b). The occurrence of cap-snatching, its contribution to viral plus-strand stability and quantitative effects on translation remain to be determined.

### The toll of LRV infection: from parasite fitness to human disease

In pathogenic microbes, signs of totivirus infection could be displayed as alterations to fitness and virulence. For example, viral presence in yeast and fungal species (Wickner, 1996; Schmitt and Breinig, 2006) offers a survival advantage through the expression of a toxin that kills their uninfected peers. Contrarily, viral infection of certain fungi (*Beauveria bassiana* and *Bostrytis cinera*) seems to reduce their virulence and thus their efficacy in the bio-control of agricultural pests (Castro et al., 2003; Dalzoto et al., 2006). More commonly, however, totiviral infections do not show significant phenotypic alterations or pathology. This, along with their widespread distribution in protists, plants, arthropods, and possibly fish suggests that Totiviruses could be more abundant than estimated. Given the increasing number of metagenomic and “pathogen discovery” projects, we may soon be able to better estimate the prevalence of *Totiviridae* and thus determine its toll on host evolution.

Thus far, only few studies have reported on the impact of *Totiviridae* on overall mRNA turnover and protein expression in the host cell. In *T. vaginalis*, the presence of a dsRNA virus causes significant differences at the protein level between infected and non-infected varieties, including quantitative and qualitative differences in cysteine proteinases known to modulate *T. vaginalis* pathogenesis (Provenzano et al., 1997). Most provocative and still to be confirmed is the up-regulation in synthesis and surface expression of the immunogen P270 (Khoshnan et al., 1994), which may impact pathogenesis in the human. Other members of the *Totiviridae* family have been described as directly pathogenic. The myonecrosis virus, for example, was shown to cause cell death in the skeletal muscle of the Pacific white shrimp (Lightner et al., 2006; Poulos et al., 2006) while a new member of the *Totiviridae* family has been implicated in Atlantic salmon cardiomyopathy syndrome (Lovoll et al., 2010). This latter virus was classified through amino acid sequence alignment of capsid-RDRP proteins, showing the closest match with the *Giardia lamblia* virus and stands as the first example of a totivirus directly infecting a member of the metazoa (Lovoll et al., 2010).

As already introduced in this review, a particularly interesting relationship exists between the dsRNA virus of *Leishmania* parasites and the human host, having important consequences on the clinical outcome of leishmaniasis. LRVs have been detected in both active and healing lesions or scars, confirming LRV presence in field isolates (Cadd et al., 1993; Salinas et al., 1996; Saiz et al., 1998; Ogg et al., 2003) although the their prevalence and clinical significance is not yet known. In *Leishmania*, the role of LRV on parasite metabolism and gene expression has not been studied in detail and there is no information on why some parasite strains are able to maintain LRV and others not. While RNAi may serve to cull the viral herd in *Leishmania* (thereby allowing their persistence through Malthusian fitness), a range of enzymes and proteases potentially able to aid viral replication can also be found in *Leishmania* parasites. A specific cysteine protease (possibly a homolog of *LmjF08.1040* according to our database search) has been implicated in the processing of the viral capsid-RDRP precursor (Carrion et al., 2003). On the other hand, the LRV capsid protein possesses an endoribonuclease that cleaves uncapped, non-polyadenylated, plus-strand, viral mRNA at a single site in the 5′-untranslated region, which was proposed to also cleave decapped, non-polyadenylated mRNAs belonging to the parasite (MacBeth and Patterson, 1995, 1998; Ro et al., 2004).

**Figure 3:**
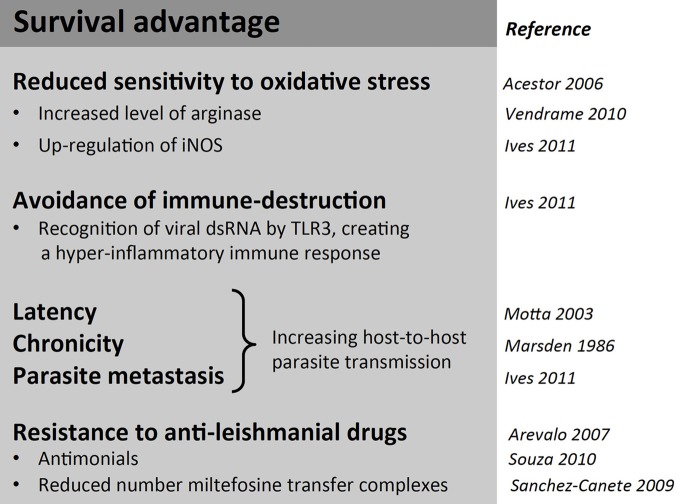
**Summary of possible survival advantages in *Leishmania (Viannia)* parasites associated with LRV infection.** Despite the metabolic and translational toll on the host, LRV infection is associated with some benefits in survival advantages for its host parasite.

While molecular studies on LRV continued more or less constantly since its description, the interest in its functional and clinical role remained low. Several groups speculated an influence of LRV presence on parasite virulence, but over 10 years passed with no major studies on the biological impact of LRV1 on *Leishmania* parasites and no efforts to analyze isogenic or clonal lines at a protein level. An interest in LRV as a determinant of virulence resurfaced only in 2011 through the use of *L. guyanensis* clones isolated from human patients (Ives et al., 2011). The isolates were previously classified by differing metastatic proclivities, ranging from highly metastatic (M+) to non-metastatic (M−) as seen in the golden hamster model (Martinez et al., 1991). In this study, they compared soluble proteomes from promastigotes and revealed that M+ and M− clones express distinct acidic and neutral isoforms of cytosolic tryparedoxin peroxidase (cTXNPx). This differential expression was conserved in *L. (Viannia)* isolates from cutaneous (M−) vs. mucosal (V+) lesions and may relate to the mechanisms by which the activity of cTXNPx is modulated and/or the gene product(s) are post-translationally modified. The metabolic role of cTXNPx in oxidative stress reiterates the importance of antioxidant defense in the development of MCL and further endorses the cTXNPx gene as a unique factor underlying the development of metastatic infection. Further differences in cTXNPx activity were viewed under oxidative stress and during infection. Upon H_2_O_2_ treatment or heat shock, cTXPNx is mostly detected in a dimerised form in M+ *L. guyanensis* and *L. panamensis* strains, while it is mostly undimerized in M− parasites. These data provide evidence that protection to the hostile, oxidative environment encountered in the host cell by *Leishmania* promastigotes could be linked to cTXPNx conformation and may be relevant to intracellular parasite survival and persistence, which are prerequisites for the development of metastatic disease (Acestor et al., 2006; Walker et al., 2006). Whether survival advantages of metastatic *Leishmania* in stressful situations (Figure 3) are due to a direct action of LRV on parasite metabolism or indirectly by an action via the host innate immune response merits further investigation.

### Maintenance of LRV infection

It is unknown how LRV1 is maintained and transmitted in the *L*. (*Viannia*) subgenus. Extracellular transmission of *Totiviridae* is rare, albeit documented for the *G. lamblia* virus, which can infect virus-free hosts (Ro et al., 1997a). Within the Totiviridae family, viral spread is either vertical (from mother to daughter) or horizontal [by cell fusion during mating and hyphal anastomosis (Dalzoto et al., 2006)]. Infection of virus-free *Leishmania* parasites has failed or lasted only transiently (Armstrong et al., 1993), a fate similarly encountered by workers studying other Totiviruses. Thus, extracellular transfer of the dsRNA virus is not likely to occur in *Leishmania*. In fungi, transmission take places during genetic exchange and may apply to leishmania (Ravel et al., 1998; Nolder et al., 2007; Odiwuor et al., 2011). It is only recently that exchange was experimentally demonstrated in *L. major* via a sexual cycle in the sand fly stage (Akopyants et al., 2009). However, unlike yeast and fungi, this form of mating (and thus viral exchange) is highly infrequent in *Leishmania* parasites, perhaps explaining the low prevalence of LRV across the genus.

Besides a single report on the generation of LRV- parasites from an LRV+ parent (Ro et al., 1997a), a stable LRV infection into naïve parasites has not yet been demonstrated. This lone observation has proven difficult to reproduce in our laboratories, and may be the result of a fortuitous event reflecting natural variation of LRV virus levels as well as its loss (Ives et al., 2011). Nonetheless, these isogenic LRV+ and LRV− strains have since been used to study the role of RNAi machinery (Lye et al., 2010) as well as that of LRV in disease outcome (Ives et al., 2011; Ronet et al., 2011). Likewise, there has been no reported success in the development an infectious system with which to stably reintroduce LRV into LRV-deficient lines. It is possible that the RNAi machinery itself is the obstacle impeding viral introduction, as subgenus *Viannia* parasites (but not “higher” Leishmania) possess a potent RNAi pathway (Lye et al., 2010). A recent study with the yeast L-A virus suggested that reintroduction of an active RNAi pathway in this species was incompatible with maintenance of the dsRNA virus (Drinnenberg et al., 2011) and correspondingly, the absence of this pathway in *S. cerevisae* could explain why they carry such taxing quantities of virus. The interplay between dsRNA viruses and RNAi has been suggested as a force contributing to the loss of RNAi in both *Leishmania* and *S. cerevisae* (Beverley, 2003; Robinson and Beverley, 2003; Drinnenberg et al., 2011). More research is needed to assess the toll of LRV presence on parasite pathogenicity and what benefits may be gained by the maintenance of such an infection.

## Immune response in MCL patients

### The Th1/Th2 dogma

**Figure 4:**
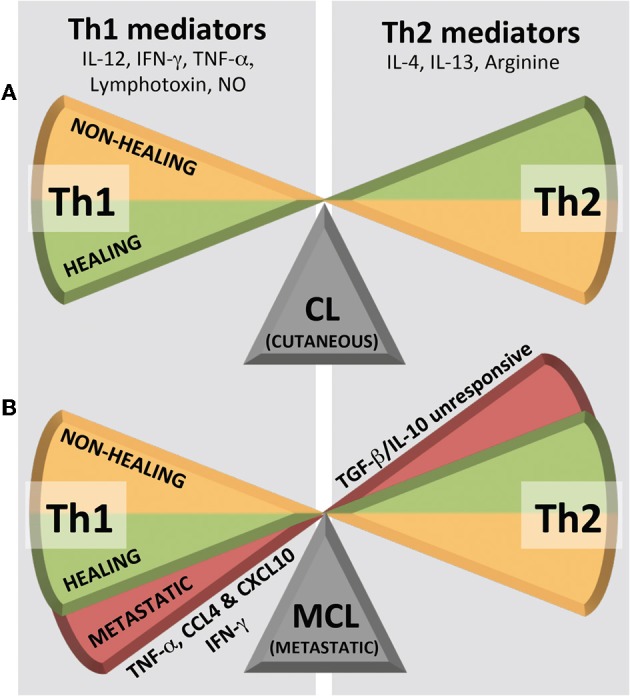
**Immunological Th1/Th2 dogma in leishmaniasis. (A)** Cutaneous leishmaniasis (CL) shows a simple, graded bipolar phenotypic range while **(B)** metastatic/mucocutaneous leishmaniasis (MCL) shows its third phenotype in a Th1 extreme. NO, nitric oxide.

In animal models of cutaneous leishmaniasis caused by *L. major*, the immunological dogma correlates resistance to disease with the development of a CD4^+^ Th1 response, and susceptibility with a CD4^+^ Th2 response (Figure 4A). Th1 cells are characterized by the production of IFN-γ and lymphotoxin whereas Th2 cells classically produce IL-4, IL-5, and IL-13. In humans and in cutaneous leishmaniasis caused by species other than *L. major*, although the protective parameters are similar, the response is not as polarized as reported in mouse models. Here, the response more graded and heterogeneous, albeit still harboring a predominant CD4^+^ Th1 type response upon healing (either after treatment or spontaneously) and having a Th2 cytokine profile of IL-4 and IL-13 in non-healing phenotypes. Protection against *L. major* has been correlated to early IL-12 production by dendritic cells and macrophages, which in turn induces IFN-γ production by Natural Killer (NK) cells and at a later point, by Th1 cells. However, the levels of IFN-γ do not always correlate with resistance, as similar levels of this cytokine were observed following leishmanization, independently of lesion development. Tumor necrosis factor alpha (TNF-α) and IFN-γ act synergistically to induce nitric oxide synthase (iNOS) in macrophages. This enzyme catalyzes the synthesis of citrulline and nitric oxide (NO) from arginine leading to the killing of intracellular amastigote parasites. Arginine, however, is also the substrate of arginase, producing the polyamines necessary for parasite growth and may even be used by the parasite in immune evasion [for a review on L-arginine metabolism and *Leishmania* infection see Wanasen and Soong (2008)]. For example, it was shown that *Leishmania* arginase could subvert iNOS-dependent killing by reducing macrophage L-arginine (Gaur et al., 2007). The Th1/Th2 paradigm further disregards the influence and interaction of other, more recently described, T-cell subsets, such as Th17, vilified as the architect of chronic destructive inflammation such as that seen in metastatic leishmaniasis, but nonetheless, has stood as the most reliable guide in the prediction of parasitotoxic immune responses.

Challenging the dogma is the response seen in MCL, where high levels of Th1 pro-inflammatory cytokines (TNF-α and IFN-γ) are associated with T cell hyperactivity and a worsening of the disease (Carvalho et al., 1985; Silveira et al., 2009). Furthermore, patients who are refractory to treatment and demonstrate an inability for their cytokine profile to switch from a mixed Th1/Th2 to a predominantly Th1 type (Pirmez et al., 1993; Diaz et al., 2002), still have elevated levels of TNF-α in their lesions. A common immunological trait in MCL is a decreased response to Th1-suppressing cytokines (IL-10 and TGF-β) (Bacellar et al., 2002). Thus, while in CL, tipping the Th1/Th2 balance in either direction results in a simple, graded, bipolar healing *vs*. non-healing phenotype (Figure 4A), MCL displays its third phenotype as a deleterious pro-inflammatory Th1 extreme (Figure 4B). This response is often not only “extreme” in its quantity of inflammatory mediators, but also in its persistence over time. In this archetypal MCL, increased levels of TNF-α, CXCL10, and CCL4 within a mixed intra-lesional Th1/Th2 response, results in an emphasized cytotoxic T cell activity, which may underlie the localized tissue damage and development secondary lesions that characterize the pathology of MCL (Pirmez et al., 1993; Faria et al., 2005; Gaze et al., 2006; Vargas-Inchaustegui et al., 2010). What pushes the host immune system into this deleterious Th1 extreme may be related to either a genetic hyperactivity within the host or, indeed, the presence of a potent innate immunogen in the parasite, such as LRV1 dsRNA.

The cytokine triggers detonating infectious metastasis might also be related to a loss of control. Indeed, as mentioned above, MCL lesions often have diminished expression of IL-10R, rendering them insensitive to its anti-inflammatory role that usually works to maintain an appropriate response by dampening the torrent of stimulatory signals in the lesion environment. Type I IFNs work in a similar manner, down-regulating IFNγ-R on the surface of macrophages and thus rendering these cells insensitive to intracellular pathogen killing via reactive nitrogen species (Rayamajhi et al., 2010). Another possible trigger may be an early peak of IFN- β. Early IFN-β in models of metastatic leishmaniasis has been reported to chronically modulate the immune response and parasite killing, favoring parasite survival and infectious dissemination to different organs [as was shown for the dissemination of *L. braziliensis* in TNF-α^−/−^ mice (Rocha et al., 2007)]. Indeed, mice deficient for the IFN-β receptor (IFNAR) were protected from leishmaniasis, presenting with smaller lesions and reduced antigen-specific and Th1 immune responses after infection with *L. amazonensis* (Xin et al., 2010). In addition, increased and sustained neutrophil recruitment in IFNAR^−/−^ mice participate to boost parasite killing. Consequently, type I interferons and molecules of its signaling pathway may emerge as therapeutic targets in infections with species of New World *Leishmania*.

### Innate immunity to *totiviridae*: TLRs, RLRs and NLRs

The innate immune response to *Leishmania* parasites has been the topic of recent reviews, which summarize TLRs (Liese et al., 2008; Faria et al., 2012; Singh et al., 2012). In the case of the *L. (Viannia)* subgenus, this has been exemplified for TLR2 in *L. panamensis* and *L. amazonensis* (Gallego et al., 2011; Vivarini Ade et al., 2011). Outside our recent description in *Leishmania guyanensis*, viral endosymbionts as innate immunogens have not yet been described. The immunogenicity of their molecular components could reveal many potential pathways to influencing the human immune response. The nucleic make-up of Totiviridae (dsRNA) is sensitively recognized by a variety of pattern-recognition receptors in immune cells. In our report, LRV recognition occurred through the TLR3 pathway. TLR3, is well-known to recognize dsRNA, producing an IFN-β mediated anti-viral response, which we proposed as underlying the exacerbated disease phenotype. Indeed, TLR3^−/−^ mice showed no LRV-mediated increase in destructive inflammation. Interestingly, its ssRNA-recognizing sister receptor, TLR7, is also stimulated by LRV components (as seen by a slight up-regulation of pro-inflammatory cytokines in *in vitro* macrophages) but did not extend to a clinical role in the *in vivo* mouse model (Ives et al., 2011; Ronet et al., 2011). The role of the last endosomal TLR recognizing DNA (TLR9) has not yet been reported on. It should be pointed out that dsRNA can also be detected by a variety of non-TLR pathways. For example, Rig-Like-Receptors (RLRs) are stimulated through binding either the 5′ RNA triphosphate (RIG-1) or whole dsRNA such as poly I:C (MDA-5) and both converge to mediate IFN-β dependent inflammation. Further, this nucleic acid is also known to stimulate the Nod-Like-Receptors (NLRs) through an unknown mechanism, sparking inflammasome activity and thus may be another mechanism of immune disruption and perhaps even local tissue damage. Whether or to what extent these RLR and NLR pathways are involved in metastatic leishmaniasis is still unknown. These innate pathways peak our interest in the primary host-parasite encounters and we predict that pattern recognition in epithelial and neutrophil cells will attract particular attention in the coming years. This potential for immunogenicity amongst totiviruses, is not only important for the disease pathogenesis of their pathogen hosts, but also serves to single-out these pathogen symbionts as unique molecular targets for use in diagnostic and therapeutic strategies.

### The oxidative response in MCL

Despite the similarity between LRVs in the *L. (Viannia)* subgenus, there are still major variations in metastatic phenotype amongst infections from different LRV+ parasite isolates. It is possible that differences in sensitivity to oxidative stress could motivate these deviations, prolonging parasite survival or triggering the latency, which underlies many recurrent infections. Several oxidative mediators could participate in *Leishmania* killing such as NO (produced by iNOS from L-arginine), H_2_O_2_ (detoxified in mammalian cells by catalase or glutathione peroxidase) and cytoplasmic superoxide as well as its byproduct, peroxinitrite, [generated mainly by NADPH oxidase at levels controlled by cytoplasmic superoxide dismutase (SOD1)]. Even though contradictory results have been reported on the roles of NO, ROI (Rocha et al., 2007; Khouri et al., 2009), and the regulation of arginase (Muleme et al., 2009), it is generally accepted that they are key mechanisms in the elimination of leishmanial infection. The same view is held for LRV-infected parasite species such as *L. guyanensis*, albeit that the role of NADPH oxidase is still matter of debate (Rocha et al., 2007). Oxidative evasion could certainly be achieved through parasitic manipulation of arginase I, a key enzyme in the production of urea (instead of parasitotoxic NO) from arginine. This enzyme is thought to be antagonized by IFN-γ (Menzies et al., 2010) and under control of Th2 cytokines (IL-4, IL-10, and IL-13) (Modolell et al., 1995). This latter regulatory mechanism has since been challenged (Muleme et al., 2009). Indeed, metastatic *L. braziliensis* species were reported to induce higher levels of insulin-like growth factor (IGF), a substance known to up-regulate arginase activity (Vendrame et al., 2010). None of these reports, however, have addressed the possible influence of LRV infection on oxidative resistance.

As previously mentioned, the recurring concurrence of resistance to oxidative stress in metastatic parasites seems more than fortuitous. Interestingly, however, these parasites also seem to induce a higher level of the oxidative stress to which they are resistant, begging the question of: which causes which? For example, the metastatic *L.g.* M5313 clones persist in activated bone marrow derived macrophages (BMMϕ) despite an elevated NO level (Acestor et al., 2006), shown to be the result of an up-regulation in the iNOS gene (Ives et al., 2011; Ronet et al., 2011). We reported that *L. guyanensis* cytoplasmic tryparedoxin peroxidase (cTXPNx) could be involved in this protection, as it seems to increase resistance to H_2_O_2_ in metastatic parasites (Acestor et al., 2006). Conversely, rapidly healing skin lesions are also associated with increased iNOS expression as well as IFN-γ, IL-12 and their signal transducer, STAT4 (Rocha et al., 2007). This latter group of molecules was described as essential in the healing of *L. major* infection, whereas only IFN-γ and STAT4 (but not IL-12) were essential in the healing of *L. mexicana* infection (Buxbaum et al., 2002).

Khouri et al. showed that the elimination of *L. braziliensis* could depend on superoxide (and not NO) (Khouri et al., 2009) and further that exogenously added IFN-β impairs superoxide killing, favoring parasite survival by up-regulating cytoplasmic SOD1 (Khouri et al., 2009). These data suggest that production of IFN-β could increase susceptibility of the host to infection [for a review see (Trinchieri, 2010)]. It is here where the presence of an intra-parasitic dsRNA virus becomes especially important in the disease process. Viral dsRNA is innately recognized by TLR3, and known to induce the secretion of IFN-β and other pro-inflammatory mediators. Already, several murine models of infection have shown that early TLR3-induced IFN-β secretion does not play its expected anti-viral role but is rather involved in the eruption of a pathological immune response. Indeed, TLR3^−/−^ mice, in these infections, have an increased survival rate when compared to their wild type (Lang et al., 2006; Le Goffic et al., 2006; Cavassani et al., 2008). Further, TLR3 ligation up-regulated pro-inflammatory mediators (such as IFN-β, TNF-α, IL-6, and various chemokines) that promoted organ damage with a high dependency on type I IFNs (specifically IFN-β), CD8^+^ cytotoxic T cell and NK cell activity (Lang et al., 2006; Le Goffic et al., 2006; Cavassani et al., 2008). Further evidence of the destructive influence of type I IFNs has been observed when blocking these cytokines *in vivo* (Sakaguchi et al., 2003; O'Connell et al., 2004; Lang et al., 2006; Baccala et al., 2007; Rayamajhi et al., 2010). These studies also demonstrate that type I IFNs modulate the Th1/Th2 polarization of the immune response via the inhibition IL-12 expression, impairing DC maturation, decreasing T cell IFN-γ production and down-regulating IFNγ-R on myeloid cells (O'Connell et al., 2004; Lang et al., 2006; Baccala et al., 2007; Cavassani et al., 2008). The importance of oxidative stress in controlling *L. (Viannia)* parasite load and its immune response is evidently not understood and studies have yet to account for the impact of *Leishmania* dsRNA virus on NO and superoxide level.

## Experimental animal models of MCL

The golden hamster is possibly the best animal model in which to study metastatic leishmaniasis caused by the *L. (Viannia) guyanensis, panamensis,* and *braziliensis* (Sinagra et al., 1997). It has been extensively used with parasites isolated from sand flies or human MCL lesions to reproduce the clinical manifestations of metastatic leishmaniasis (Travi et al., 1988). Differences in disseminative propensity were found between various species and individual strains (Martinez et al., 1991). In particular, an infective strain of *L. (Viannia) guyanensis* (WHI/BR/78/M5313) isolated from a sand fly was shown to be highly metastatic. Cloned lines of this strain, however, also showed a graded metastatic ability in the hamster. Thus, allowing for the characterization of highly (M+), moderately and non-metastatic (M−) parasites within the same clone. These graded phenotypes remained stable over several passages (Martinez et al., 2000) and were caused by varied intensities in inflammatory response (Travi et al., 1996). A decade later, we acquired these same strains, finding the LRV^high^/LRV^low^ parallel to their M+/M− capability (Ives et al., 2011): the correlation forming the basis of this review. Since then, a different *L. guyanensis* strain (M4147) derived from a human lesion has also shown metastatic ability in the hamster model (Rey et al., 1990) and we were able to achieve an identical metastatic relationship between LRV+/LRV− clones.

Although the development of secondary lesions is rarely seen in murine models of MCL, the LRV-based virulence of these parasites seems to be reflected in an increased disease severity at the primary site of infection (Ives et al., 2011; Ronet et al., 2011). Other reports have shown varying results: in BALB/c mice, for example, M4147 *L. guyanensis* did not induce progressive lesions (Sousa-Franco et al., 2006) and only very low number of parasites could be recovered at the site of infection. In these footpads, there was a well-preserved inflammatory cell population and intact tissue architecture. In the same model, *L. panamensis* parasites behave like most *Leishmania* species inducing a non-healing footpad swelling (Rojas et al., 1993; Goto et al., 1995) having the expected cytokine profile of a “losing battle” in the draining lymph nodes. Here, an early induction of IFN-γ and minimally detectable IL-4 at 24 h post infection was followed by a complete reversal 7 days post infection (Guevara-Mendoza et al., 1997). Whether these strains were infected by LRV is unknown but thus far, LRV has not been identified in *L. panamensis* (although as noted earlier, this species has been less thoroughly screened). Regardless, it is likely that the presence of LRV in *L. guyanensis* and *L. braziliensis* strains is only one of the mechanisms contributing to disseminated and metastatic leishmaniasis.

Our study on the TLR-dependent recognition of LRV in the *L. guyanensis* M5313 strain was continued in TLR3^−/−^ and TLR7^−/−^ murine models. As anticipated, the lack of TLR3 significantly decreased footpad swelling and diminished parasite load whereas no distinguishable difference in disease phenotype was observed in mice infected with M- (LRV1^low^) parasites or between WT and TLR7^−/−^ infected mice with either parasite isolate. We could conclude that these TLR3-dependent responses to M+ (LRV1^high^) parasites resulted in elevated disease severity in mice and provide evidence that LRV1 within metastasizing *L. guyanensis* parasites promotes inflammation, and is implicated in susceptibility to infection. Our mouse work correlates LRV1 presence in parasites with the hyper-inflammatory immune responses characteristic of MCL disease. This is the first description that TLRs can contribute to *Leishmania* susceptibility (and not resistance). It remains to be determined how representative the mouse model is of the involvement of LRV in disease pathology in humans. Although the current mouse model does not display all of the hallmarks of MCL, it provides many advantages over the hamster, specifically in the broader availability of knockout varieties and reagents for an immune response that is much better described.

*L. braziliensis* has greatly differing phenotypes in either animal model. In the hamster, it was shown that secondary visceral lesions could arise from a primary CL and also that CLs could appear subsequently to primary visceral ones (Almeida et al., 1996). While infections in BALB/c mice only gave rise to small, transient footpad lesions (Dekrey et al., 1998; Lima et al., 1999) and further, require a high dose inoculum (1 × 10^7^) to generate this weak response. Similarly in an ear dermis model of *L. braziliensis* (De Moura et al., 2005) [based on an existing model in *L. major* (Belkaid et al., 1998)], lesions were minor and temporary (De Moura et al., 2005), albeit that parasites persisted in the draining lymph nodes (De Moura et al., 2005; Rocha et al., 2007). The differences in disease outcome may be formed at the level of the immune response. IFN-γ produced by CD4+ and CD8+ T cells was shown to be important (Dekrey et al., 1998; De Souza-Neto et al., 2004; De Moura et al., 2005; Rocha et al., 2007) concomitant with the expression of a broad spectrum of chemokines attracting neutrophils, monocytes/macrophages, NK, CD4+, and CD8+ T cells (De Moura et al., 2005; Teixeira et al., 2005). It is also interesting to note the Th2 cytokine IL-4 was only present at low levels during the first 3 weeks of *L. braziliensis* infection, becoming undetectable from day 42 post infection (Dekrey et al., 1998).

The factors underlying the presence or absence of metastatic lesions in animal models are, as yet, unknown. For the human, however, it has been suggested that quiescent or slow-growing parasites could be reactivated and metastasize to mucocutaneous sites following immuno-suppressive treatment (Motta et al., 2003) or during in stress situations (Travi et al., 1988, 1996) which are perhaps not encountered in the laboratory model.

## Host genetic factors in MCL

At the genetic level in humans, alleles encoding TNF-α, TNF-β, IL-6, CXCR1, and CCL2/MCP1 were associated with an increased relative risk of MCL (Cabrera et al., 1995; Nashleanas et al., 1998; Castellucci et al., 2006, 2010; Ramasawmy et al., 2010). Some examples of polymorphisms identified in patients with MCL include (1) a homozygous polymorphism in intron 2 of TNF-β, (2) a single base pair substitution at position -308 in the promoter for TNF-α, and (3) a single G-to-C base pair substitution at position -174 in the promoter for IL-6 (Blackwell, 1999; Sakthianandeswaren et al., 2009). Additionally, in Venezuelan MCL patients, there is a preferential expression of HLA class II DQw3 (Lara et al., 1991).

The presence of LRV and its dsRNA acting on TLR3 could shed light on other possible genetic polymorphisms in the host. The roles of TLR3 in the metastasis of carcinomas has already been described, where it was shown to both prevent as well as underlie metastatic processes and their tropism to nasopharyngeal tissue. Here, TLR3 activation inhibited metastasis via down-regulation of the chemokine receptor CXCR4 (Zhang et al., 2009) but has since been shown to have an opposing role, promoting metastasis (Matijevic and Pavelic, 2011). Similarly, it is also possible that the effect of LRV can be differentially modulated further downstream of its TLR3-dependent recognition, for example in the induction of IFN-β and production of CCL5, CXCL10, IL-6, and TNF-α. The balance between pro- and anti-inflammatory cytokines and chemokines can be shifted by polymorphisms in *TLR* genes (Kutikhin, 2011), increasing the risk of chronic inflammation and infection. If a predisposition to the disease can be funneled down to single immune mediators, it may pave the way for a new, immunomodulatory approach to the treatment and prevention of metastatic leishmaniasis.

## Current therapies

Current therapeutic strategies in leishmaniasis are far from satisfactory. The growing demand for new anti-leishmanial drugs is parallel to a spreading drug resistance across the genus as well as their variable efficacy and toxicity in different patients (Croft et al., 2006). A major problem exists in the immunocompromised population, often co-infected with HIV and following an already toxic regimen of hepatically metabolized treatments. The most popularly used drugs in the management of leishmaniases are based on the pentavalent antimonials, sodium stibogluconate (Pentostam), and meglumine antimoniate (Glucantime) (Harder et al., 2001). Amphotericin B, a polyene antibiotic, has been used as a second-line treatment for leishmaniasis since the 1960s. It is proposed to work by encouraging a Th1 immune response and inducing cytokines such as TNF-α and IL-1β as well as generating a parasite-killing respiratory burst (Wolf and Massof, 1990; Vonk et al., 1998; Cuna et al., 2007).

MCL development is, so far, impossible to predict and exceedingly difficult to treat; often displaying drug resistance and predisposing the host to opportunistic infections. Patients are frequently refractory to antimony treatment and in extreme cases, a combination of antimony and amphotericin B is required but even then, treatment failure is observed. Although antimony resistance has been proposed to cause these relapses, the factors underlying MCL development and its subsequent treatment failure are unknown. Sensitivity to antimony treatment might also depend on intrinsic factors within the infecting *L*. *(Viannia)* parasites (Arevalo et al., 2007; Souza et al., 2010). Whatever the case may be, the limited efficacy and major side effects of antimony treatment expose a clear need for the development of new drugs specifically designed to treat MCL. These drugs should take into account the intrinsic variations of the metastatic parasite as well as the complex inflammatory processes shown to be the root cause of this outcome.

Indeed, controlling inflammation could be an alternative to complement conventional drug therapies. Already, interesting results have been reported for the use of the anti-inflammatory drug tamoxifen in MCL patients (Miguel et al., 2009). Further, treatment with the anti-inflammatory TNF-α inhibitor, pentoxyphylline in combination with antimony was shown to be effective in MCL patients unresponsive to antimonial therapy alone (Lessa et al., 2001). Other immunomodulatory drugs have been since proposed such as thalidomide (Blackwell, 1999). However, anti-inflammatory drugs in leishmaniasis should be used with caution, especially when there is no evidence of hyper-inflammation. This is because anti-inflammatory or immunosuppressive agents can result in the reactivation of leishmaniasis as seen in leishmanial patients treated with anti-TNF-α for rheumatoid arthritis (Franklin et al., 2009).

Miltefosine, an oral drug effective against VL has been tested in MCL. Surprisingly, *L. braziliensis* parasites were more resistant to miltefosine than *L. donovani* (Sanchez-Canete et al., 2009). This difference could be explained by a reduced transport of the drug through the miltefosine transport complex (Sanchez-Canete et al., 2009); a situation illustrating the importance of the intrinsic factors of metastatic parasites in determining the efficacy of drug therapies. Other parasite parameters, which could influence the efficacy of anti-leishmanial drugs, are the variable levels of resistance to oxidative stress and strain-specific differences on the innate immune response. Antimony has been shown to activate the cell death pathway in several *Leishmania* species by generating oxidative stress in the form of H_2_O_2_ and NO (Mehta and Shaha, 2006). It was recently confirmed that cTXPNx (an enzyme known to detoxify oxidative compounds), plays a crucial role in protecting *L*. *donovani* parasites against H_2_O_2_ and also by counteracting antimony drug response (Iyer et al., 2008). Furthermore, increased resistance to NO in human isolates of *L. (Viannia)* can be correlated to larger lesions (Giudice et al., 2007) as well as underlie the poor responsiveness to antimony therapy (Souza et al., 2010). Thus, there is a great need to improve upon the disappointing arsenal of drugs for mucocutaneous leishmaniasis (MCL), which are currently poorly suited to the widely variable metabolic and immunological abilities of metastatic parasites.

## Concluding remarks

The intracellular parasites of the *Leishmania (Viannia)* subgenus harbor a unique risk for infectious metastasis and the development of complicated and difficult-to-treat secondary lesions. This risk was shown to have roots in both intrinsic parasite factors as well as in the immune response launched by the host, where a hyper-inflammatory over-reaction destroys local tissue and influences the efficacy of anti-leishmanial drugs. MCL is a common outcome of parasite metastasis, forming debilitating secondary lesions in the mucosa of the mouth and nose where inflammation accounts for much of the morbidity associated with the disease (Marsden, 1986; Martinez et al., 1991, 1992; Osorio et al., 1998; Herwaldt, 1999). Although heritable polymorphisms have been identified in both the MCL host and parasite, the genetic, and epigenetic factors predisposing an *L. (Viannia)* infection to metastatic complications have not yet been investigated in great detail.

Our recent data has emphasized the role of an intrinsic parasite factor in the devolution of disease *i.e*., *Leishmania* dsRNA virus that, when present in *L guyanensis*, acts as a potent innate immunogen, redirecting the immune response of the host by inducing a hyper-inflammatory reaction and possibly triggering dissemination (Ives et al., 2011; Ronet et al., 2011). Although it is likely that LRV is not the only factor involved, its presence could explain differences in the clinical outcomes observed between *Leishmania* species and/or strains and holds great potential as a new target for treatment strategies. Therapeutic possibilities exist in either pursuing LRV itself (antiviral therapy) or in reversing the anti-viral immune response it induces. Indeed, drugs countering the type of hyper-inflammation caused by LRV have been successful in the treatment of MCL. Tamoxifen (Miguel et al., 2009) and a TNF–α inhibitor, pentoxyphylline (Lessa et al., 2001) for example, were used in combination with antimony and were shown to aid in the resolution of disease. It would be interesting to determine whether these drugs have an independent or supporting role to antimony, perhaps only working to create an environment in which antimony is effective. Refractory and secondary MCL lesions often display antimony resistance and drugs reverting this process are obviously much desired.

The possibility that MCL development is caused by a misguided immune response against its viral hyperpathogen provides a novel means of diagnosing the metastatic risk of leishmaniasis as well as creating a better understanding of the treatment needed to cure it. The fact that this risk could be caused by a parasitic factor (as opposed to a human susceptibility) is not surprising, as it would follow the many observations that deviations in parasite phylogeny mirror their clinical ones.

This is the case between evolutionarily distant members (Old World *vs*. New World) as well as between different isolates of the same strain: differences, which are echoed in their immunogenicity. Comparing the LRV-mediated process of metastasis with existing models of parasite dissemination could elucidate the mechanisms underlying recurrence and reactivation, thus creating a much-needed model system of metastatic leishmaniasis.

The discovery of LRV as an innate immunogen altering the course of leishmaniasis should motivate further investigation on the toll of such viral hyperpathogens on other infections. In the case of metastatic leishmaniasis, it may provide us with a unique opportunity to intervene at a clinical level: for the first time, enabling the diagnosis of metastatic risk and providing a unique target for future therapeutic approaches.

### Conflict of interest statement

The authors declare that the research was conducted in the absence of any commercial or financial relationships that could be construed as a potential conflict of interest.
